# Knowing One’s Place: Parental Educational Background Influences Social Identification with Academia, Test Anxiety, and Satisfaction with Studying at University

**DOI:** 10.3389/fpsyg.2017.01326

**Published:** 2017-08-03

**Authors:** Stefan Janke, Selma C. Rudert, Tamara Marksteiner, Oliver Dickhäuser

**Affiliations:** ^1^Department of Psychology, University of Mannheim Mannheim, Germany; ^2^Department of Social Psychology, University of Basel Basel, Switzerland

**Keywords:** first-generation students, social identity, test anxiety, satisfaction with studying, social class, higher education

## Abstract

First-generation students (i.e., students whose parents did not attend university) often experience difficulties fitting in with the social environment at universities. This experience of personal misfit is supposedly associated with an impaired social identification with their aspired in-group of academics compared to continuing-generation students (i.e., students with at least one parent with an academic degree. In this article, we investigate how the postulated differences in social identification with the group of academics affect first-generation students’ satisfaction with studying and test anxiety over time. We assume that first-generation students’ impaired social identification with the group of academics leads to decreased satisfaction with studying and aggravated test anxiety over the course of the first academic year. In a longitudinal study covering students’ first year at a German university, we found that continuing-generation students consistently identified more strongly with their new in-group of academics than first-generation students. The influence of social identification on test anxiety and satisfaction with studying differed between groups. For continuing-generation students, social identification with the group of academics buffered test anxiety and helped them maintain satisfaction with studying over time. We could not find these direct effects within the group of first-generation students. Instead, first-generation students were more sensitive to effects of test anxiety on satisfaction with studying and vice versa over time. The results suggest that first-generation students might be more sensitive to the anticipation of academic failure. Furthermore, continuing-generation students’ social identification with the group of academics might have buffered them against the impact of negative experiences during the entry phase at university. Taken together, our findings underscore that deficit-driven approaches focusing solely on first-generation status may not be sufficient to fully understand the importance of parental educational background for students’ well-being. More specifically, continuing-generation students might reap benefits from their parental educational background. These benefits widen the social gap in academia in addition to the disadvantages of students with first-generation status. In sum, understanding the benefits of continuing-generation status has important implications for interventions aiming to reduce social class gaps in academia.

## Introduction

In the past, Western universities had a long tradition of being elitist institutions. Even at the beginning of the 20th century, they still opened their gates almost exclusively to white men from higher social classes (Thomas et al., 1979). Since then, many western countries have adopted educational policies aiming for educational expansion and the step-wise inclusion of women, members of lower classes as well as of different ethnicities into universities (Schofer and Meyer, 2005; Housel and Harvey, 2009). While this policy seemed to create equal opportunities, members of these newly included groups often had to overcome strong obstacles because of a lack of financial as well as cultural capital that helps to succeed in academic environments (Pascarella et al., 2004; Engle and Tinto, 2008; Stebleton and Soria, 2012). Nevertheless, the proportion of students from a low social class, with no family background in academia and/or an ethnicity deviating from the respective privileged ethnicity has increased over the past decades. This can be interpreted as a decrease in institutionalized discrimination (Housel and Harvey, 2009; Hauschildt et al., 2015). However, psychological research shows that despite this improvement, students from social groups that are traditionally and continuously underrepresented at university are still likely to experience subjective feelings of detachment at universities (e.g., Walton and Cohen, 2007, 2011). Among others, this applies to *first-generation students* (i.e., students whose parents did not attend university), who seem to experience difficulties fitting in with the social environment of universities compared to *continuing-generation students* with at least one parent who attended university (Stephens et al., 2012b). These difficulties may be explained using a *social identity* approach (Tajfel and Turner, 1986; Ellemers and Haslam, 2011). First-generation students’ enrollment at university can be understood as striving for social mobility from lower social classes of origin to the *aspired high-status in-group of academics*. The high importance of this venture for first-generation students may evoke threat that is rooted in a perceived *personal misfit* to the group of academics (Stephens et al., 2012b, 2015). As a consequence, first-generation students may struggle to integrate the social category of “being an academic" into their social self. Eventually, these difficulties may result in insecurities regarding one’s personal ability to cope with academic challenges.

In the present study, we propose that first-generation students’ social identity influences their test anxiety and satisfaction with studying. In contrast to previous cross-sectional or experimental studies on personal well-being of first-generation students, we investigated how these effects unravel over time by using longitudinal data. We focused on the first academic year in which first-generation students are introduced to the world of their new aspired in-group of academics for the first time. During this time, first-generation students are confronted with new social norms and unfamiliar educational standards. In contrast to continuing-generation students, first-generation students cannot rely on their parents for an explanation of these new rules. Thus, they might feel less familiar with the new setting than continuing-generation students and experience difficulties to align their self with their aspired in-group and, thus, to academia. Eventually, first-generation students might experience uncertainty regarding their personal academic capabilities, which may result in aggravated test anxiety and impaired satisfaction with studying.

### Social Identity and the Self of First-Generation Students

Our assumptions regarding first-generation students’ social identity are based on *Self-Categorization Theory*, a micro-theory within Social Identity Theory (Tajfel and Turner, 1986; Brewer and Gardner, 1996). Self-Categorization Theory suggests that the self contains intrapersonal (e.g., personality traits) and interpersonal aspects (e.g., social roles, group memberships) that influence how individuals perceive themselves and the world around them. Furthermore, Self-Categorization Theory states that group identification in particular influences personal self-esteem through social comparisons of important in-groups with out-groups. Thus, individuals who identify themselves with a low status group (e.g., uneducated workers, oppressed minorities) will experience a drop-in self-esteem when they compare themselves to higher-status groups. As a result, individuals will aim to improve their self-esteem which can be achieved either through social competition (collective striving for group superiority), social creativity (redefining the group or changing the dimension of social comparison) or social mobility (escaping the devalued group; Ellemers and Haslam, 2011). The last strategy is particularly important for first-generation students who strive to leave groups characterized by lower education to become part of the community of academics. When individuals engage in a new social group with high importance for their future life, it is essential for them to figure out whether they can identify with this aspired in-group or not. To do so, individuals compare their own characteristics and values with allegedly prototypical characteristics and values of the group to evaluate whether they fit in with that group (Brewer and Gardner, 1996). Perceiving a personal misfit with an aspired social in-group impairs the inclusion of the in-group into the self, which has been labeled *social identity threat* (Ellemers and Haslam, 2011; Stephens et al., 2012b).

When first-generation students consider their own fit with the aspired in-group of academics, they may come to the conclusion that their lack of a parental background in academia still distances them from this group, because the group of academics still largely consists of people from highly educated families (Housel and Harvey, 2009; Hauschildt et al., 2015). Our assumption that parental education is important for the social identity of first-generation students aligns well with research that has shown that the personal educational background constitutes a key component of the social self (Ethier and Deaux, 1994; Thomas and Azmitia, 2014). We think that the educational background is even more important for the social self than the parental income level, which has also been used to identify students’ social status (Ostrove and Long, 2007). Empirical evidence indeed suggests that one’s educational background is more closely associated to lifestyle, behavior and psychological functioning than income (Snibbe and Markus, 2005; Stephens et al., 2007, 2012a). While parental income can provide students with information on the question of whether they belong to an elite classified by money, we assume that it is the educational background of their parents which signals to students whether they fit in with the aspired in-group of academics.

It should be noted that effects of the parental education level are not limited to students’ education prior to university. Research shows that first-generation students have higher retention rates (Ishitani, 2003) and are less likely to enroll in master and PhD programs even after they successfully finished their undergraduate studies (Jaksztat, 2014). This is likely because first-generation students experience a misfit between their personal values and the norms prevalent in academic contexts (Stephens et al., 2012a,b). More specifically, while universities emphasize agentic norms (e.g., pursuing one’s own career), first-generation students often have strong communal values and motives (e.g., helping their community of origin). Stephens et al. (2012b) used the term *cultural mismatch* to describe this normative divergence between university and individual, which eventually diminishes personal identification with the aspired in-group of academics. These findings support the notion that education in school or even at university does not eliminate the influence of the parental education on students’ behavior and psychological functioning.

In sum, we assume that first-generation university students experience a stronger misfit regarding the group of academics when they enter university compared to continuing-generation students. The experience of detachment is supposedly stable throughout the first year at university, because students’ family background does not change over time. Moreover, while personal values may eventually shift in times of educational transition (Krishnan, 2008), communal values are still very likely to be reinforced by family and peers from the community of origin. Thus, these values are probably less likely to attenuate in favor of agentic values because individuals with high communal values find it highly important to stay in contact with their original community. Further, and in line with college impact models (Terenzini et al., 1996; Strayhorn, 2006), we assume that the experienced mismatch will affect first-generation students’ personal experiences and well-being at university. More specifically, we assume that personal misfit to the group of academics raises doubts regarding one’s own capabilities to succeed in academia. In the long run, we would expect that first-generation status eventually reduces satisfaction with studying and may lead to increased test anxiety. This is because the personal ascendance to the aspired in-group of academics supposedly depends on meritocratic principles (i.e., the ability to perform well in classwork, tests and exams). First-generation students who doubt their personal fit with academia may also doubt their personal ability to cope with such challenges, which eventually results in aggravated test anxiety. Supporting this assumption, research finds that first-generation status can have a negative effect on the emotional state and the cortisol level of students when they are confronted with personal misfit prior to performing an academic task (Stephens et al., 2012b). We assume that the negative effects of social identity threat on test anxiety and satisfaction with studying are most pronounced during the first semester when students move physically as well as mentally from their prior social environment to their new academic environment and identity-based questions are thus most prevalent.

### Current Research

Research on the negative effects of first-generation status was mainly conducted in the laboratory (Stephens et al., 2012b) or with applied cross-sectional designs (Reay et al., 2001; Ostrove and Long, 2007). Consequently, we neither know how these effects unfold over time nor whether they remain stable in the field. Thus, the main goal of our research is to investigate longitudinal effects of first-generation status on social identification with the group of academics. Moreover, we are interested in the effect of first-generation status on test anxiety and satisfaction with studying. We assume that first-generation students experience stronger detachment from their aspired academic in-group compared to continuing-generation students starting from the very beginning of their studies. Furthermore, we hypothesize that this initial group difference in social identification with the aspired in-group of academics eventually impacts the level of test anxiety and satisfaction with studying over the time span of the first semester. We propose that students who initially struggled to identify with their new in-group of academics also develop more test anxiety and less satisfaction with studying after the first semester. Additionally, we assume that first-generation status indirectly affects this development through the degree of social identification with the group of academics. We also hypothesize that first-generation status still indirectly influences social identification with the group of academics, satisfaction with studying and test anxiety at the end of the first year at university. This assumed indirect effect should be due to initial group differences in social identification with the in-group of academics and construct stability after the end of the transition phase to university (i.e., the first semester).

## Materials and Methods

We conducted a quantitative longitudinal study at a public German university with a focus on social and economic sciences. About 11.000 students are enrolled at this university, which can be considered an average university size in the context of the German higher education system. It should be noted that, different from the United States system, students do not have to pay tuition fees in Germany and university education is typically partitioned into three subsequent stages (bachelor, master, doctorate). We questioned students at the very beginning of this educational process: during their bachelor studies. Students answered three online surveys over the course of their first year at university: during the first month of their first semester, six months later (after their first semester), and an additional six months later (after their first academic year). The online survey was distributed by the administration of the university to all students enrolled in bachelor programs starting in Fall 2013. Participation in the study was voluntary and informed consent was obtained for all participants via online consent forms that were embedded into all three surveys. Every participant had to agree to the following statement: “I hereby confirm that I am of age, that I have read the consent form, and that I agree to take part in this study under the described conditions”. Participants were assured that they could quit the questionnaire at any time and that all of their responses would remain confidential. The study was conducted in full accordance with the Ethical Guidelines of the German Association of Psychologists (DGPs) and the American Psychological Association (APA). By the time the data was acquired, it was also not customary at the respective university, nor at most other German universities, to seek ethics approval for survey studies on social identity and well-being. The study exclusively makes use of anonymous questionnaires. The three separate questionnaires were matched for the longitudinal analyses by relying on codenames. No identifying information was obtained from participants. We had no reasons to assume that our survey would induce persisting negative states (e.g., clinical depression) in the participants.

### Sample

A sample of 536 students (*M_age_ =* 20.0 years; *SD* = 2.6; 65.3 percent female) completed the online survey at the beginning of their studies. This corresponded to 23.3 percent of freshmen at the university in question in the year 2013 (2304 freshmen in total). 36.8 percent of the participants (*n* = 193) were classified as first-generation students (no parents with an academic degree), while the rest of the sample was classified as continuing-generation students (at least one parent with an academic degree) based on students’ self-reported parental education level. This percentage stayed relatively constant over time (33.9 percent first-generation students after the first semester; 38.2 percent first-generation students after the first academic year; χ^2^(2) = 0.42, *p* = 0.812). Students from all undergraduate programs that the university offered were represented, that is, business, economics, humanities, social sciences, law, information technology, and mathematics. At time point two, 322 students completed the survey and 249 completed the survey at time point three^1^.

### Measures

#### Social Identification

**FIGURE 1:**
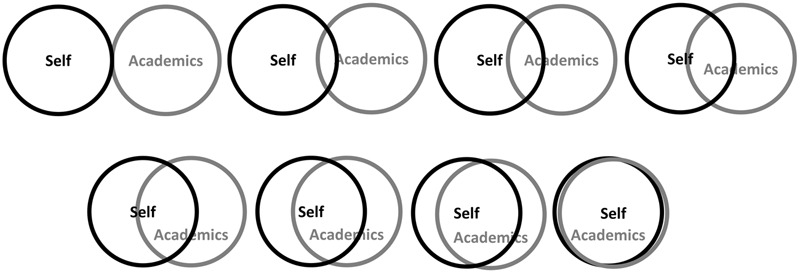
Visualization of the used measure for social identity. The participants were asked to choose the pair of circles that best reflects their sense of belonging to the group of “academics”.

Identification with the group of academics was measured with a modified German version of the *inclusion of the in-group in the self measure* (Aron et al., 1992; Tropp and Wright, 2001). The scale consisted of eight pairs of circles which differed in the degree of their overlap (1 = no overlap; 8 = full overlap). The first circle was labeled “self” and the second one was labeled “academics” (see **Figure 1**). The participants were asked to choose the pair of circles that best reflected their belongingness to the group of academics^2^.

#### Satisfaction with Studying

Satisfaction with studying was assessed with the short form of a well-validated German self-report scale (Fragebogen zur Studienzufriedenheit; Westermann et al., 1996, 2002). The scale consists of three subscales, i.e., satisfaction with the content, with the course conditions, and with the ability to cope with study load. Each subscale had three items. A sample item from the subscale measuring satisfaction with the content is *“Overall, I am pleased with my academic experiences.”* We combined all nine items into one general score as recommended by Westermann et al. (1996). Negatively worded items were reverse-coded. The overall scale acquired an acceptable reliability at time points two and three (α_t2_ = 0.85/α_t3_ = 0.84). Because students had just enrolled at university and would have had difficulties answering some of the items (e.g., “*I am feeling tired and stressed while studying.*”), we only used five of the original nine items (α = 0.68) to assess satisfaction with studying at time point one (see recommendations by Hiemisch et al., 2005).

#### Test Anxiety

Test anxiety was measured with the corresponding subscale of the Achievement Emotions Questionnaire in German wording (AEQ; Pekrun et al., 2011). The subscale consisted of 12 items. A sample item is “*Before exams I feel nervous and uneasy.*” We aggregated all items to one overall score for test anxiety (α_t1_ = 0.92/α_t2_ = 0.91/α_t3_ = 0.92) as recommended by Pekrun et al. (2011).

### Analyses

In the first set of analyses, we conducted a cross-lagged panel analysis to investigate direct and indirect effects of first-generation status on students’ social identification with the group of academics, their satisfaction with studying, and their test anxiety. We allowed direct paths from first-generation status (coded 0 = continuing-generation student; 1 = first-generation student) on all three outcome variables at time point one. Potential influences of initial group differences in the high-school GPA (“*Abiturendnote”* in the German education system) were controlled for.

In addition, we investigated the influence of all three constructs on each other over time while controlling for stability. While we assumed that all stability coefficients would reach significance due to self-stabilizing processes, we expected the cross-paths of social identification with the group of academics on the other two constructs to be statistically significant only between time points one and two (critical transition period).

Lastly, we investigated whether first-generation status indirectly influenced the three outcome variables at time points two and three. We assumed that first-generation status would indirectly influence satisfaction with studying and test anxiety after the first semester via social identification at the beginning of ones’ studies. These indirect effects were expected to indicate the negative long-term effects of the initial struggle for social identification within first-generation students. We also assumed that first-generation status would indirectly affect social identification at time points two and three as well as satisfaction with studying and test anxiety at time point three.

In a second set of analyses, we tested whether the obtained longitudinal relationships were generalizable between groups or whether group membership (first- vs. continuing-generation status) would moderate these relationships. For this purpose, we tested the invariance of the path structure (generalizability of the path model to both groups) as well as invariance of the path coefficients (generalizability of path coefficients to both groups). We calculated Chi-Square difference tests to investigate these levels of invariance. Thereby, we assumed that the observed longitudinal effects would merely reflect different group levels of social identity threat for first-generation students compared to continuing-generation students. Thus, we expected invariance for the model structure as well as the path coefficients.

All structural equation models were conducted with Mplus Version 7.2 (Muthén and Muthén, 1998–2012). We applied the robust MLR-estimator and relied on the guidelines given by Schermelleh-Engel et al. (2003) when investigating the model fit. Therefore, we distinguished between an acceptable model fit (*RMSEA* ≤ 0.08, SRMR ≤ 0.10, *CFI* ≥ 0.95) and a good model fit (*RMSEA* ≤ 0.05, SRMR ≤ 0.05, *CFI* ≥ 0.97). Missing data were handled with the Full Information Maximum Likelihood Imputation provided by Mplus.

## Results

Means, standard deviations and zero-order correlations between all scales are depicted in **Table 1**. A closer look at the zero-order correlations suggests negative associations between first-generation status and social identification with the in-group of academics at all three time points. Furthermore, social identification seems to be negatively related to test anxiety and positively related to satisfaction with studying in most instances.

**Table 1:** Zero order correlations, descriptives, and internal consistencies.

	*M*	*SD*	α	(1)	(2)	(3)	(4)	(5)	(6)	(7)	(8)	(9)
**Time Point 1**												
(1) First-generation status	0.63	0.48	–									
(2) Social identification	4.57	1.96	–	–0.23^∗∗^								
(3) Test anxiety	2.87	0.96	0.92	0.12^∗∗^	–0.24^∗∗^							
(4) Satisfaction with studying	3.98	0.74	0.68	–0.02	0.18^∗∗^	–0.35^∗∗^						
**Time Point 2**												
(5) Social identification	4.69	1.88	–	–0.30^∗∗^	0.69^∗∗^	–0.23^∗∗^	–0.14^∗^					
(6) Test anxiety	2.88	0.81	0.91	0.12^∗^	–0.29^∗∗^	0.73^∗∗^	–0.35^∗∗^	–0.27^∗∗^				
(7) Satisfaction with studying	3.68	0.69	0.85	–0.12^†^	0.29^∗∗^	–0.35^∗∗^	0.62^∗∗^	0.25^∗∗^	–0.43^∗∗^			
**Time Point 3**												
(8) Social identification	4.74	1.81	–	–0.26^∗∗^	0.57^∗^	–0.14^∗^	0.18^∗∗^	0.66^∗∗^	–0.20^∗∗^	0.25^∗∗^		
(9) Test Anxiety	2.81	0.84	0.92	0.10	–0.17^†^	0.64^∗∗^	–0.31^∗∗^	–0.11^†^	0.79^∗∗^	–0.38^∗∗^	–0.13^∗∗^	
(10) Satisfaction with studying	3.59	0.70	0.84	–0.11^†^	0.12	–0.22^∗∗^	0.57^∗∗^	0.10	–0.36^∗∗^	0.77^∗∗^	0.18^∗∗^	–0.39^∗∗^

*First-generation status is a dichotomous variable, with 0 = continuing-generation status and 1 = first-generation status. The scales measuring satisfaction with studying and test anxiety ranged from 1 (no agreement) to 5 (strong agreement). The item measuring social identification with the group of academics ranged from 1 (no overlap self/academics) to 8 (full overlap self/academics). ^†^*p* < 0.10; ^∗^*p* < 0.05; ^∗∗^*p* < 0.01.*

**FIGURE 2:**
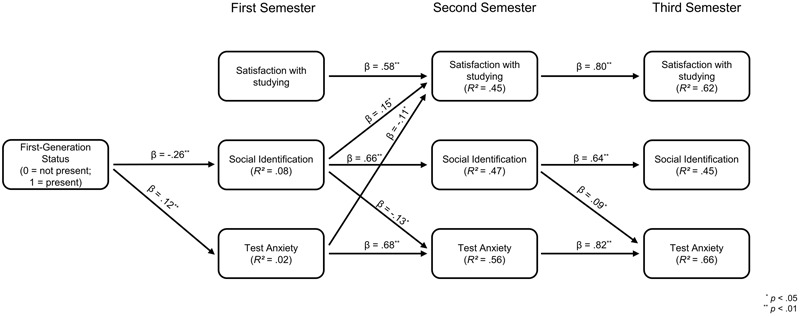
Initial cross-lagged panel model. All undireceted paths were excluded from the figure for better comprehensibility as well as all paths that did not reach significance. Significant negative correlations occurred for test anxiety and satisfaction with studying at all three time points (*r_T1_* = –0.34, *p* < 0.001; *r_T2_* = –0.21, *p* = 0.001; *r_T3_* = –0.36, *p* < 0.001). Furthermore, social identification was significantly negative associated to test anxiety (*r* = –0.19, *p* = 0.002) and significantly positive associated with satisfaction with studying (*r* = 0.18, *p* = 0.001) at time point one. All effects of first-generation status on outcome variables are controlled for possible influences of the GPA (*Abiturnote*).

Our initial cross-lagged panel model fitted the data well, χ^2^(21) = 59.35, *p* < 0.001, *CFI* = 0.96, *RMSEA* = 0.06, *SRMR* = 0.04. All statistically significant path coefficients are depicted in **Figure 2**. As expected, we found a direct effect of first-generation status on social identification with the group of academics. This effect indicated that first-generation students identified with the aspired in-group of academics to a lesser extent than continuing-generation students at the beginning of their studies. Moreover, the hypothesized cross-paths from social identification at time point one on the degree of test anxiety as well as on satisfaction with studying at time point three reached significance in the postulated direction. The more students identified with the group of academics at the beginning of their studies, the more they experienced satisfaction with studying and the less they experienced test anxiety at the end of their first semester. Besides these postulated effects, the data also revealed two unexpected significant paths. First, we found a positive direct effect of first-generation status on test anxiety, indicating that first-generation students already experienced aggravated test anxiety at the beginning of their studies compared to continuing-generation students. However, the effect was rather small, which was indicated by the insignificant degree of explained variance on test anxiety (*R*^2^ = 0.02; *p* = 0.171). Second, we found a direct effect of social identification at time point two on test anxiety at time point three in an unexpected direction. More specifically, we found that the more students identified themselves as academics after their first semester, the more they experienced test anxiety at the end of their first academic year.

We found that first-generation status had negative indirect effects on social identification with the group of academics (β_indirect_ = –0.17; *p* < 0.001) and satisfaction with studying (β_indirect_ = –0.04; *p* = 0.027) at time point two through initial group differences in social identification. First-generation status also positively influenced test anxiety at time point two through initial group differences in social identification (β_indirect_ = 0.04; *p* = 0.020) and test anxiety (β_indirect_ = 0.08; *p* = 0.010). We also found that first-generation status indirectly affected social identification (β_indirect_ = –0.11; *p* < 0.001), test anxiety (β_indirect_ = 0.03; *p* = 0.021), and satisfaction with studying (β_indirect_ = –0.03; *p* = 0.027) at time point three via initial group differences in social identification and construct stability in the outcome variables at time point three as we had anticipated. Significant total effects in the same direction supported all indirect effects of first-generation status on social identification and test anxiety. The total effects of first-generation status on satisfaction with studying were considerably lower and only marginally significant at time point two (β_total_ = –0.09; *p* = 0.054) or even nonsignificant time point three (β_total_ = –0.05; *p* = 0.203). However, the fact that these observed total effects did not reach conventional significance on a *p* < 0.05 level could reflect the reduction of statistical power due to drop-out over the course of the longitudinal study.

Lastly, we investigated whether the previously obtained path structure was moderated by group membership. Therefore, we investigated whether the previously obtained path model as well as the path coefficients were invariant for first- and continuing-generation students. The analyses indicated that the structural model was applicable for both groups; Δχ^2^(9) = 11.54, *p* = 0.240. However, further investigations revealed that both groups were not identical when it comes to the values of the obtained path coefficients; Δχ^2^(36) = 67.55, *p* = 0.001. Thus, we calculated a multi-group to investigate how the path coefficients differed between first- and continuing-generation students; χ^2^(18) = 41.54, *p* = 0.001, *CFI* = 0.98, *RMSEA* = 0.07, *SRMR* = 0.03. The results of the multi-group model are described into more detail in the following two paragraphs^3^.

### Path Model for Continuing-Generation Students

**FIGURE 3:**
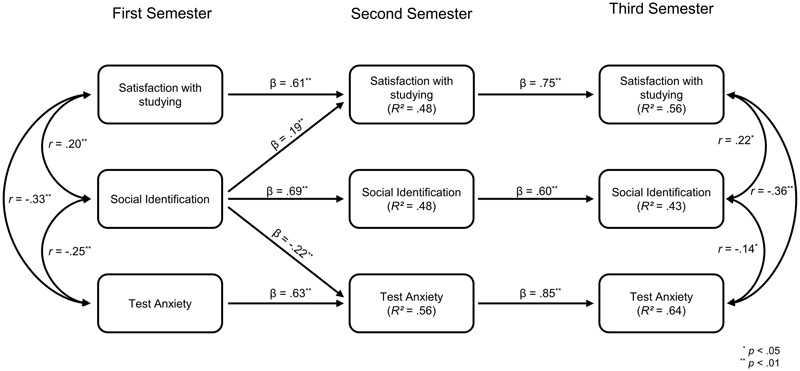
Conducted cross-lagged panel model in the subgroup of continuing-generation students. Paths that did not reach significance were excluded for better comprehensibility.

The path coefficients for continuing-generation students are depicted in **Figure 3**. The path model in this group seemed highly similar to the original single-group model. That being said, the cross-paths of social identification with the group of academics on satisfaction with studying and on test anxiety between time points one and two were considerably larger than in the single-group model^4^. We also did not find the unexpected positive effect of social identification with the group of academics on test anxiety after the first academic year that we found in the single-group model (see above).

### Path Model for First-Generation Students

**FIGURE 4:**
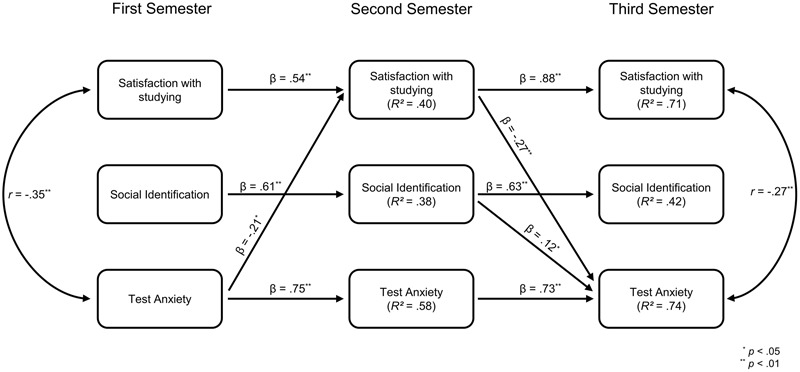
Conducted cross-lagged panel model in the subgroup of first-generation students. Besides the depicted paths, three undirected paths reached significance at time point two: Satisfaction with studying was positively associated with social identification (*r* = 0.39, *p* = 0.001), while test anxiety was negatively associated with social identification (*r* = –0.33, *p* = 0.004) as well as with satisfaction with studying (*r* = –0.27, *p* = 0.001). These undirected paths were excluded from the figure for better comprehensibility as well as all paths that did not reach significance.

Next, we took a closer look at the path model in the subgroup of first-generation students. The path coefficients for this subgroup are depicted in **Figure 4**. These path coefficients differed largely from the original single-group model. None of the cross-paths from social identification on test anxiety and satisfaction with studying reached significance in between time points one and two. Instead, we observed a statistically significant cross-path from test anxiety on satisfaction with studying in this time-period. This path was negative, suggesting that stronger test anxiety at the beginning of one’s studies led to less satisfaction with studying after the first semester for first-generation students. Additionally, a negative cross-path linked satisfaction with studying at time point two to test anxiety at time point three. This means that satisfaction with studying after the first semester reduced the degree of test anxiety after the first academic year in the group of first-generation students. We once again obtained the unexpected positive cross-path between social identification measured at time point two on test anxiety measured at time point three that had not reached significance in the group of continuing-generation students.

## Discussion

In the present study, we demonstrated that continuing-generation students identified themselves more strongly as academics than first-generation students when they enrolled at university. Furthermore, social identification with the group of academics influenced test anxiety and satisfaction with studying over time. More specifically, a higher degree of social identification led to lower test anxiety and higher satisfaction with studying after the first semester as well as after the first academic year. These effects cannot sufficiently be explained by individual differences in the performance level, since we controlled for initial academic abilities (measured using high-school GPA) between first- and continuing-generation students in our structural equation models. The proposed model structure was more pronounced in the group of continuing-generation students than in the group of first-generation students: Initially, we had assumed that first-generation students would struggle to identify as academics at the beginning of their studies, which would eventually result in increasing test anxiety and decreasing satisfaction with studying. However, we exclusively found this postulated path structure in the group of continuing-generation students. In the group of first-generation students, we found significant cross-paths of test anxiety on satisfaction with studying and vice versa instead. In the following section, we discuss how this surprising finding may be explained by a positive effect of social identification through entitlement in continuing-generation students as well as anxiety in first-generation students.

### Family Background as a Buffer for Negative Experiences: Observed Effects of Entitlement

In contrast to our initial expectations, our results seem to suggest that the effect of social identification is more prominent in continuing-generation students than in first-generation students. It is possible that those students frequently had contact with other academics in the past and, thus, saw academics as a natural in-group before they even attended university. As a result, they might have developed a feeling of entitlement. This feeling of entitlement may have helped continuing-generation students cope with the challenges of their first semester: The more they identified with the group of academics, the higher was their satisfaction with studying and the lower their test anxiety over time. However, the question remains how this sense of entitlement might act as a support for continuing-generation students. One possible explanation could be that continuing-generation students are able to buffer typical negative experiences (e.g., fitting in with the new environment; first academic tests) at the beginning of their studies by reminding themselves that, despite any failures, they belong in academia after all.

### Family Background and Anxiety: Plausible Effects of Academic Uncertainty

Originally, we had assumed that first-generation students would suffer from impaired well-being as a consequence of their struggle to identify with the group of academics. However, based on the obtained data, it appears more likely that continuing-generation students actually benefit from their social identity in terms of enhanced satisfaction with studying and reduced test anxiety. Still, there are some hints suggesting that first-generation students’ social identity might lead to negative effects in a more indirect way. Taking a closer look at the pattern of results, it became evident that test anxiety plays an important role for the well-being of first-generation students. Even though the initial group differences in test anxiety are rather small, we find that test anxiety has a stronger influence on first-generation students’ satisfaction with studying over time and vice versa than for continuing-generation students. This might reflect a more distal effect of social identity. Possibly, first-generation students’ well-being might be more vulnerable to personal anxieties regarding academic failure than continuing-generation students’ well-being. This effect could be rooted in uncertainty regarding one’s own abilities to cope with academic challenges. Such an explanation would be in line with research demonstrating that individuals suffering from belonging uncertainty (i.e., uncertainty about whether they belong in their new social environment) are more likely to experience negative emotions in this new environment (Brown et al., 2007; Cockshaw and Shochet, 2010; Cockshaw et al., 2013).

### Practical Implications

Our results once again support the notion that effects of social disparities do not end when individuals enroll themselves at institutions of higher education. Specifically, our results show that initial differences in social identification lead to ongoing differences in satisfaction with studying and test anxiety, possibly through feelings of entitlement in continuing-generation students. Thereby, the applied longitudinal approach extends research on the subject matter beyond cross-sectional (Reay et al., 2001; Ostrove and Long, 2007) and experimental studies (Stephens et al., 2012b). This allows us to identify critical time spans for the development of social identification and well-being at university and, thus, possible onset points for interventions: We observed initial group differences between first- and continuing-generation students in social identification at an early point in time, a few weeks after their first semester had started. These initial differences remained stable during the following two semesters. Therefore, one can assume that interventions targeting social disparities in students’ well-being would be most effective either during a student’s very first days at university or even earlier, in the last year at school. Furthermore, the effect of social identification on students’ satisfaction with studying and test anxiety was most prominent between the first and the second semester. Indirect interventions that aim to reduce the effects of disparities on students’ well-being might thus be most effective during the first semester.

So far, most of the existing interventions aiming to reduce social-class gaps in higher education have focused on deficits of underrepresented students in social identification (Stephens et al., 2012a,b) or social belonging (Walton and Cohen, 2007, 2011). However, a deficit-driven approach might not be enough, given that our results indicate that continuing-generation students seem to profit from a head start in social identification, possibly due to personal feelings of entitlement. These students might rely on their family background as a buffer for negative experiences that occur at the beginning of their studies. Since first-generation students have no background in academia and therefore lack this buffer, they might still experience more test anxiety and less satisfaction with studying than continuing-generation students even when they participated in interventions that focus on the negative effects of their heritage. Thus, intervention programs should be supplemented with modules that focus on providing first-generation students with resources to cope with negative experiences at the beginning of their studies. This might help to reduce attrition rates in the population of first-generation students (for further recommendations on future interventions, see also Stephens et al., 2015).

### Limitations

There was a substantial number of drop-outs over the course of our study which was at least partially linked to satisfaction with studying. In particular, students who were at least somewhat satisfied with their study conditions at the beginning of their studies were more likely to continue to participate. It is highly plausible to assume that unsatisfied students might have decided to quit their studies altogether or at least changed their university during the timespan of our study. Since we were only able to survey the students currently enrolled at the university, we had no possibility to get any information on those students who quit their studies altogether. It would be interesting for future research to get more insight on the impact of the effects of social identification on the probability of dropping out of university. However, the fact that we obtained long-term effects nevertheless speaks for the robustness of these results, and the observed small to medium effect size might underestimate the actual effect sizes. Thus, our study can be considered as a conservative test of the long-term effects of students’ social identification in their first semesters at university.

Additionally, we have exclusively focused on the importance of family educational background for students’ social identity in our analyses. Other personal characteristics of freshmen may also influence whether they identify with the aspired in-group of academics. Basically, any personal characteristic that deviates from a prototypical representation of university students, who have predominantly been white males from upper social classes for centuries, may undermine students’ identification. Thus, gender, ethnicity, and social class can also be assumed to constitute students’ social identity. These factors are entangled with each other, making additive effects likely. Researchers refer to students who are characterized by a combination of variables like first-generation status, low social status or underrepresented ethnicity as doubly disadvantaged students (Jack, 2014). Doubly disadvantaged students often struggle even harder with their new place in academia than students that are characterized by only one of these variables (Harackiewicz et al., 2016). Thus, potential joint effects of first-generation status and ethnicity or income level on the social identity of freshmen seem to be a fruitful topic for future research.

However, it is important to keep in mind that “singularly disadvantaged” students also experience difficulties fitting in with academia. Our research supports the notion that one’s personal identity at university can depend on very elementary factors such as the educational background of one’s parents. This is also in line with a growing body of empirical evidence that stresses the unique importance of the educational background (Ethier and Deaux, 1994; Snibbe and Markus, 2005; Stephens et al., 2012a; Thomas and Azmitia, 2014).

Finally, it should be noted that our sample is not representative for the whole population of first- and continuing-generation students since it consisted of students who took part in our study voluntarily. Thus, our data should not be interpreted in isolation but rather under consideration of previous studies as a contribution to the growing body of evidence on the importance of family background for psychological functioning at university.

### Future Directions

It should be noted that we conducted our research at a public German university. While we do think that the described institution is representative for the German higher education system, the results of our studies might not generalize to institutions providing higher education in other countries. Even though the German higher education system is typical for western higher education systems, there are some national specifics, such as the often highly competitive selection procedures for restricted graduate programs and the absence of student tuition. Another difference to other educational systems might be that the social and educational background affects educational attainment more strongly than ethnicity (Kristen and Granato, 2007). In contrast, ethnicity seems to be a very important predictor for first-generation students’ educational attainment in the United States (Nuñez and Kim, 2012; Strayhorn, 2014). While we did investigate effects of first-generation status rather than effects of ethnicity in our study, it might be interesting to investigate a possible interaction of both factors in cross-cultural research. Cross-cultural research would also be necessary to investigate whether the obtained effects are generalizable across different cultures. For instance, one could imagine that cultural moderators like the segregation of social classes or the social status of academic titles in society could affect the observed relationships. In addition, it would also be interesting to investigate a longer time-period stretching from the first to the last semester and addressing student attrition.

Moreover, one interesting yet unexplained finding of our study is the positive direct effect of social identification on test anxiety from time point two to time point three in first-generation students. This path suggests that first-generation students that have started to associate themselves with the group of academics experience emotional strain as their studies progress. We can only speculate about the reasons for this observed effect. It is possible that the effect reflects the fear of losing the newly acclaimed in-group of academics due to perceived lack of academic abilities for first-generation students who started to affiliate with their new social environment. This explanation is in line with Jury et al. (2015) who argued that successful first-generation students have to anticipate double detachment in cases of future failure. These students are supposedly already detached from their social group of origin since they successfully integrated themselves into a new social environment with different norms than their prior social environment (Stephens et al., 2012b). Academic failure would lead to detachment of first-generation students from their new in-group of academics, because failure would indicate that they cannot keep up with the internal standards of this group. Consequently, successful first-generation students might experience test anxiety and emotional strain. Our results might show this possible downside of successful integration attempts by first-generation students. Still, we have to be cautious in interpreting the obtained effect since it was not expected and the path coefficient is rather small. Thus, it could also be a methodological artifact. However, under the condition that future research can replicate the effect, it would be an interesting endeavor to investigate the proposed explanations more closely.

## Conclusion

The presented study provides new evidence that the effects of social disparities do not end at the doorstep of university. Our results suggest that the negative consequences of social disparities nowadays might not only be due to discrimination by others or differences in personal resources. Instead, social disparities might have a more-subtle effect via individuals’ social identity. It might be a difficult task for practitioners in higher education to close this social gap. However, we tend to remain optimistic that our research contributes to the enduring goal to expose social disparities in the educational system and hope that future research can benefit from our findings when it comes to the development of interventions aiming to reduce such disparities.

## Author Contributions

All listed authors contributed meaningfully to the paper. SJ, SR, and TM developed the study concept. All authors contributed to the study design. SJ, SR analyzed and interpreted the data. SJ prepared the draft manuscript, and SR, TM, and OD provided critical revisions. All authors approve the final version to be published, and agree to be accountable for all aspects of the work in ensuring that questions related to the accuracy or integrity of any part of the work are appropriately investigated and resolved.

## Conflict of Interest Statement

The authors declare that the research was conducted in the absence of any commercial or financial relationships that could be construed as a potential conflict of interest.

## Footnotes

1 We conducted step-wise cox proportional hazard regression models to investigate the drop-out over time. The results indicated that the students who remained in the sample at the beginning of their third semester reported a slightly higher satisfaction with studying at the beginning of their studies then those who had dropped out over time (*b* = -0.22, *p* = 0.007). It is likely that this systematic drop-out effect reflects the tendency of unsatisfied students to change university or quit studying altogether over the course of time. This systematic drop-out effect was only pronounced in continuing-generation students, but not in first-generation students. Aside from this effect, we could not observe any other systematic drop-out effects based in demographics or other model variables. Thus, we can conclude that the attrition rate does not substantially distort our results pattern. Nevertheless, it still affects the statistical power for investigating the long-term effects of the obtained variables. In other words, we had a better chance to uncover long-term effects between the first and the second semester than between the second and the third semester. Power analyses indicate that the power to detect a small effect of *f^2^* = 0.09 was 0.99 for both time-spans. However, the power to detect small effects of *f^2^* = 0.02 would have been 0.72 for the time span between time point one and time point two and only 0.60 for the time span between time point two and time point three.

2 While all participants completed the described measure at time points two and three, only half of the participants were randomly assigned to complete this measure at time point one. The other half completed another version of the measure in order to test the validity of different variants. In the second version, we asked the participants to identify the circle that indicated their belongingness to the group of “children of academics” at best. However, this measure did not sufficiently address students’ social identification with the group of academics since the result pattern indicated that it merely assessed their parental educational background. Therefore, we decided to solely rely on the measure directly centering on the group of academics and did not consider data from the other half of the participants regarding social identification at time point one. We conducted a Multivariate Analysis of Variance (MANOVA) to assess whether any group differences regarding test anxiety as well as satisfaction with studying occurred between those who had and those who had not filled out the finally applied measure at time point one. We could not observe any differences between the two groups; *Hotelings’ T* = 0.000, *F*(2,533) = 0.035, *p* = 0.982, η < 0.001. Hence, we can assume that all associations between social identification measured at the time point one and other outcome variables are representative for the entire sample.

3 It would have been interesting to investigate the measurement invariance of the applied questionnaires with latent multi-group models. Unfortunately, this was not possible partly due to the reliance on single-item measures and partly due to the restricted sample size (especially at time points 2 and 3). However, the internal consistency coefficients were similar between groups for most scales (Δα < 0.03) with the singular exception of satisfaction with studying at time point 3 (Δα = 0.09). This supports the assumption that the groups interpreted the scales in the same way.

4 We used the test for the equality of path coefficients by Paternoster et al. (1998) to assess whether the obtained path coefficients differed significantly. We found that the cross-path from social identification on test anxiety was indeed significantly bigger in the subgroup of continuing-generation students than in the single-group model (*z* = –55.47, *p* < 0.001). This was also true for the path from social identification on satisfaction with studying (*z* = –43.29, *p* < 0.001).
